# Evaluation of a modular scalable system for silver-ager located in assisted living homes in Austria – study protocol of the ModuLAAr ambient assisted living project

**DOI:** 10.1186/1471-2458-14-736

**Published:** 2014-07-21

**Authors:** Christian Siegel, Barbara Prazak-Aram, Johannes Kropf, Michael Kundi, Thomas Dorner

**Affiliations:** 1 AIT Austrian Institute of Technology, Department of Health and Environment, Biomedical Systems Division, Viktor-Kaplan-Strasse 2/1, Wiener Neustadt 2700, Austria; 2 Medical University of Vienna, Centre for Public Health, Institute of Social Medicine, Kinderspitalgasse 15/1, Vienna 1090, Austria; 3 Medical University of Vienna, Institute of Environmental Health, Centre of Public Health, Kinderspitalgasse 15, Vienna A-1090, Austria

**Keywords:** Ambient assisted living, Quality of life, Evaluation, Independent living, Assistive technologies, Information and communication technology, Usability, Technology acceptance

## Abstract

**Background:**

To cope with the upcoming demographic change, economic efforts in the European Union are undertaken to promote activities in research and development of Ambient Assisted Living (AAL) solutions. As a result, a large variety of AAL products will be available in the next years. Only very few of these products are comprehensively evaluated regarding different aspects of quality of life in the target population. The aim of this study is to examine the effects of AAL on quality of life, health and technology acceptance of people at advanced age living in assisted living homes providing them the ModuLAAr Ambient Assisted Living system.

**Methods/Design:**

A treatment group of adults aged 60 years and older will be recruited within the participating assisted living homes. At baseline, the participating volunteers will report on quality of life, subjective health and sociodemographic conditions. After system installation, prospective follow-up (1, 4, 12 and 18 months) with additional reporting on technology acceptance and usability of the system will be conducted. Mixed methods data collection, linking quantitative data to interview-gathered qualitative data will be applied. Primary outcome measure will be the change in quality of life and subjective health across study duration.

**Discussion:**

As there is currently very little evidence that AAL solutions can contribute to improved health and the particular dimensions of quality of life in elderly persons, there is a need to assess these technologies and services more carefully. This field trial seeks to investigate the most relevant aspects connected to advanced information and communication technologies and their impact on daily life of residents in assisted living conditions.

**Trial registration:**

ClinicalTrials.gov: NCT02130102.

## Background

The demographic change in the next centuries was identified as one of the biggest challenges for our society and economy by the World Health Organisation (WHO). The WHO reported that in 2025 there will be more than 1.2 billion people aged 60 years and more in the world. In 2050 there will be about 2 billion elderly people. To cope with the challenges which arise with the number of elderly subjects, the WHO recommended structured political actions to enable healthy and active ageing which have to be undertaken by governments and international organisations [1].

The European Commission also declared that the ageing population will have major impact on the productivity and economic growth of its member states [2]. The amount of people aged 65 years or more will increase from 17.4% in 2010 to 29.5% in the year 2060 which could lead to a doubling of the ratio of dependent elderly (people aged 65 and above relative to those aged 15-64) [3]. This change will result in a reduced care giver availability who can provide assistance and care to old people [4,5]. To cope with these aspects, the European Commission initialized the Ambient Assisted Living Joint Program(AALJP) [6]. The assumption of this program is that some aspects of the upcoming challenges can be addressed by information and communication technologies (ICT). The most important aspect of this approach is that ICT can help elderly people to improve quality of life (QoL), increase health and live independent for a longer time in their familiar surroundings. The way the AALJP seeks to promote activities for research regarding these technologies is usually summarized as Ambient Assisted Living (AAL) solutions [6]. That kind of socio-technical solution usually provides a huge variety of technical products and services. It could reach from smart home technologies to sensor based activity and health monitoring, telemedicine and telecare as well as security and emergency call systems. This is why these solutions may have extensive positive effects on individuals, the society, and economic and medical stakeholders [7]. Within the European Research Framework Programs, the European Union and its member states have invested more than 1 billion Euros for research and innovation in the field of AAL and will continue within the research program Horizon 2020 [6,8].

In the last years, the technology driven research approach focused on developing those solutions. This is why AAL-associated products are usually evaluated according to their technical feasibility. The impact on the end-users health and QoL, the user acceptance of the solutions and the usability of product were not yet taken into account sufficiently [7]. Recent activities in funding programmes show that these aspects will be investigated in upcoming large scale pilot projects. One of these field trials is the ModuLAAr project [9] which is conducted in eastern Austria. This study will investigate how this provided module based AAL solution improves elderly’s QoL, subjective health, technology acceptance and end-user usability. This is the first large scale study in Austria which investigates the proposed effects of AAL in a long-term field trial. It will add important information to already existing outcomes of other projects and future testing regions of AAL systems regarding experiences and outcome parameters.

## Methods/Design

### Overview

ModuLAAr is a research project approved and funded by the benefit-program of the Austrian Research Promotion Agency (FFG) and the Austrian Federal Ministry of Transport, Innovation and Technology (BMVIT). The main objective of the project is to equip up to 50 flats with an AAL solution that will be adapted according to the individual needs of the residents in assisted living homes. Those already existing homes as well as new built ones will be considered for integration of the different AAL-modules. The new buildings are located in 3 and the existing homes in 6 municipalities. People living in assistive living homes provided by the Samariterbund Burgenland Rettung und Soziale Dienste GmbH may be considered for participation within the project.

### Study design

**Figure 1:**
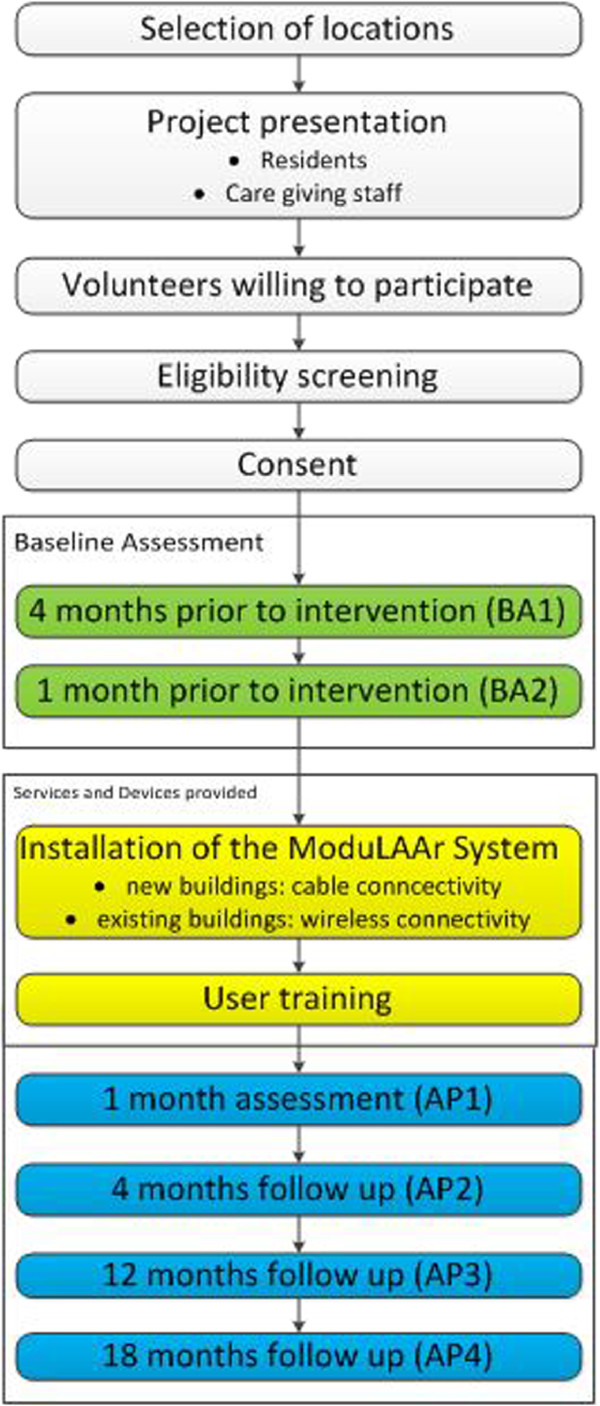
Study design and assessment points.

To establish a practical study design for the given framework of the whole project, a quasi-experimental longitudinal study with pre- and post-tests is conducted to evaluate the impact of the different AAL modules within the study population. For the study 50 persons aged 60 and older are recruited who will receive the module-based AAL solution. Those people are not necessarily dependent elderly, they live in the assisted living accommodations with an option to get support of service and nursing staff when needed or wanted. The effects of ModuLAAr are evaluated at 1-, 4, 12 and 18-months follow-ups by comparing the quantitative and qualitative results to the outcome of 2 baseline assessment points. An overview of the study design and the assessment points is provided in Figure 1. Beside the requirements of the project´s consortium members the heterogeneous combination of technical appliances, which are adapted according to the housing conditions of the participants, the creation of a control group is not appropriate to evaluate potential impacts on the study population of this specific project.

The study protocol was provided to the responsible Ethics Committee of the KRAGES Burgenländische Krankenanstalten Ges.m.b.H. who declared that for this research project it is not necessary to file an application for ethics approval. Furthermore, the Ethics Committee of the Medical University of Vienna was asked for application who responded that, according to the confirmation of the responsible ethics committee of the KRAGES, no further steps for ethics approval will have to be done. The participants´ approval will be obtained by informed consent which compiles with the Declaration of Helsinki [10].

At the beginning of the study, all eligible participants are provided with in-depth information about the study and asked to sign the informed consent form. The protocol was registered at clinicatrials.gov (Identifier: NCT02130102).

### Recruitment and eligibility

After consortiums’ decision about the participating assisted living home locations, the project is presented to the residents and - if residents want to - their relatives by one or more sessions called ‘Info Cafés’. Due to the circumstances that some assisted living homes are connected to a local nursing home, also the associated social service staff will be introduced to the project. During the project presentation, the involved persons have the opportunity to ask questions about the provided solutions and to critically reflect eventual occurring prejudices regarding the applied technologies and services.

The volunteers who declare that they are willing to take part in the study are screened for eligibility. This study aims to recruit up to 50 residents aged 60 years and above who meet the inclusion criteria.

Inclusion criteria:

1. Male or female residents of an assisted living home of the Samariterbund Burgenland Rettung und Soziale Dienste GmbH

2. Aged 60 years and older

3. Able to provide informed consent

4. Nursing level ≤ stage 4

Exclusion criteria:

1. Mini mental state examination score < 17 at baseline assessment point 1 (BA1)

2. Not able or unwilling to complete questionnaires

3. Relevant health threatening event during study which avoids further participation

4. Death of the resident

Those who meet the inclusion criteria are asked to subscribe the informed consent provided by the investigators to participate in the project.

### Study objectives

The study aims to evaluate the provided ambient assisted living solutions by measuring the changes in QoL, subjective health and technology acceptance. The primary objective of this study is to increase the domain specific QoL of the involved residents by this intervention, measured with a mixed-method approach which takes into account quantitative as well as quantitative information.

It is proposed to increase social integration due to the provided technical solutions which will have impact on interaction with other residents and relatives. Further objective of the study is to improve resident´s knowledge of the individual health status and thereby have positive effects on health behavior.

Furthermore the results shall identify the key facts how technology acceptance for AAL solutions can be enhanced and influenced.

### ModuLAAr system

The system is based on a flexible and scalable home server architecture named Homer Platform [11] developed by the AIT Austrian Institute of Technology GmbH. User interface are tablet computers with a pre-loaded software application. Furthermore, a software platform will be provided to share data between the residents and their relatives or social services.

To address the building conditions, the new built homes are equipped in construction phase with a cable bound infrastructure and a centralized installation of the server; already existing houses will be adapted with wireless technologies and decentralized installation of mini-computers.

**Figure 2:**
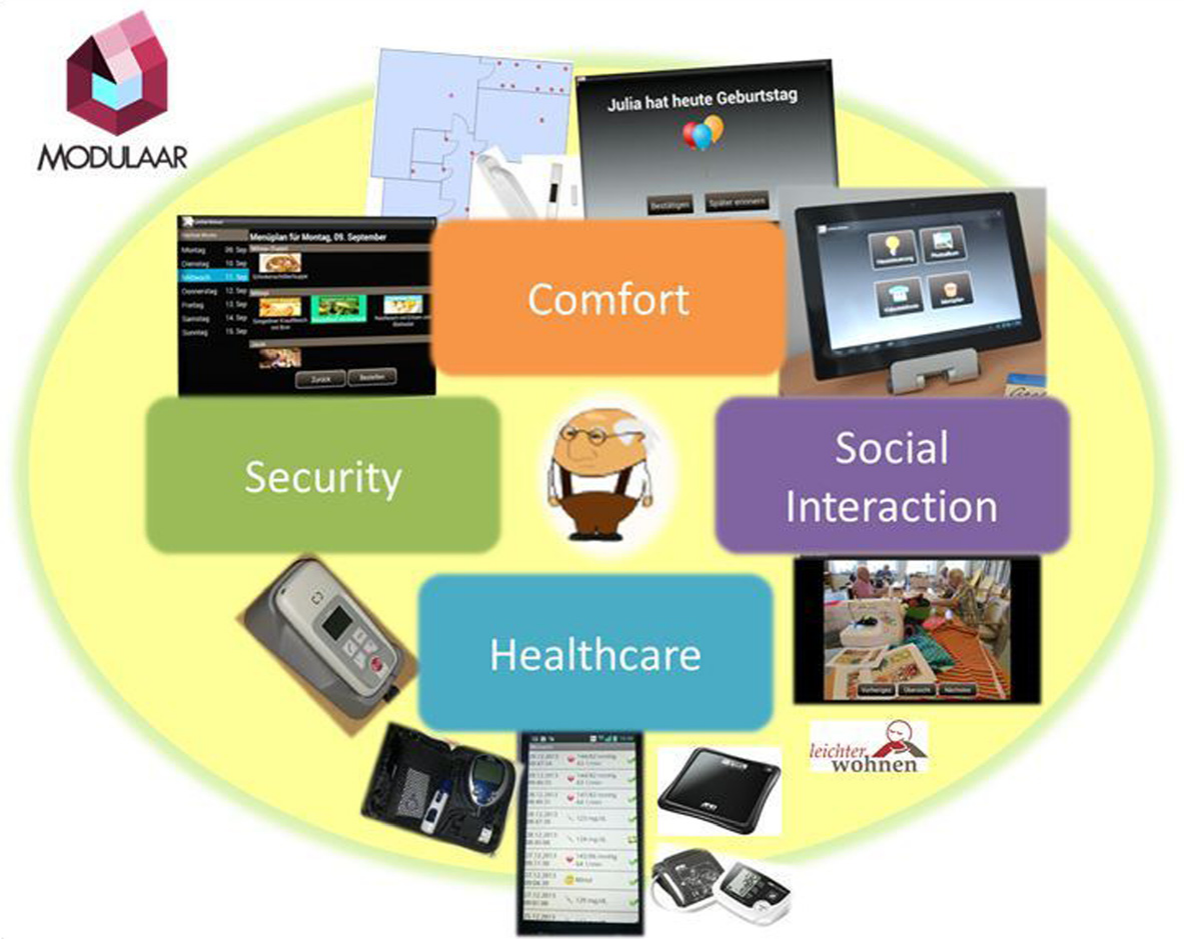
The ModuLAAr Ambient Assisted Living System.

The ModuLAAr system consists of technologies and services which are applied in four module-domains (Figure 2) which correlate with the health and QoL domains which shall be impacted [9]:

1. Social Interaction

2. Comfort

3. Security

4. Healthcare

### Social interaction

To enhance social interaction with relatives and the service provider, a video telephone, pre-installed on the tablet computer, will be provided. Additionally, a photo sharing platform is integrated in the system that enables residents to review photos on the tablet computers. The data can be administered by the study participants (contacts for video telephone, photos), social service staff or relatives if access is granted by the resident.

### Comfort

Smart home technologies are installed in the new buildings to monitor and control windows, doors, stove and light conditions. The existing houses are equipped with wireless sensors that enable status monitoring of the windows and doors.

To enhance comfort, there are reminder functions which appear on the tablet computer when appropriate. Messages are generated if defined calendar entries, e.g. birthdays, appointments or other relevant actions (e.g. medicine intake) have to be accomplished. The reminder functions can be administered by the residents, service provider or relatives who have access for using the software platform.

This module also involves a meal ordering system which enables easy access to provided menus of the social service provider. This function is limited to the homes that are connected to a nursing home with meal compartment.

### Security

The bathroom surveillance system is based on the installed movement detectors and software algorithms that calculate if a relevant event (e.g. falls) could have been occurred and sends automatically an emergency call to the defined alarm partner. The residents have the opportunity to get an emergency call system for their home and for outdoor activities (a mobile emergency call system) to manually send an emergency message if needed.

The security module provides warning messages of household equipment (e.g. to keep an eye on an active stove) and status information of the applied smart home technologies (e.g. close the door before going to bed).

### Healthcare

Participants receive a healthcare package, which is based on NFC-gadgets (near field communication devices). The device to collect health related data is a smart-phone with a pre-loaded software application developed by the AIT. The connected gadgets are blood pressure monitors, weight scales and blood glucose meters. The software application also provides the possibility to report the actual health conditions (levels: good, average and bad).

If the participants want to share their collected health-related data (e.g. blood pressure) with relatives, care service provider or medical doctors, they have the possibility to use this telemedicine-function for monitoring of data-trends.

### Intervention

After baseline assessment (BA1 and BA2), the participants are provided with the different ModuLAAr modules based on their individual interests and housing conditions (e.g. also residents without diabetes are provided with blood glucose meter if wanted; old buildings are equipped with smart home monitoring technology – the ability to control windows or doors is not provided in already existing old buildings). The installed devices will remain in the homes until the projects’ end. After installation, extensive initial system training takes place in the home of the resident. Our aim is that the enrolled study participants have the opportunity to use the system for at least 12 months until the project ends. Within study duration, a follow-up training session after 2–4 months, depending on the individuals’ need for training, is planned. Depending on the point of time of recruitment there will be up to 4 assessment points where technology acceptance and usability will be reviewed. All questionnaires are completed in the course of individual interviews with the residents to address their individual deficiencies if necessary (e.g. the participant is not able to fill in the questionnaires due to cognitive limitations). After the project is completed, the tablet computer will remain property of the resident.

### Measurements

The study participants are evaluated at 6 points in time: baseline assessment at 4 months (BA1) and 1 month before installation (BA2), 1 month after installation (AP1), after 4 months (AP2), after 12 months (AP3) and if applicable after 18 months or otherwise on project ending (AP4). Not each measurement is used on every point in time. To identify developing trends before intervention the data related to subjective health and QoL between the two baseline assessments (BA1, BA2) are compared to the equal time span of the two assessments points after installation (AP1, AP2). Table 1 summarizes the planned measurements at several times (BA1, BA2, AP1, AP2, AP3, AP4) in the study population.

**Table 1:** Questionnaires and qualitative assessment methods in ModuLAAr

	**BA1 (−4 months)**	**BA2 (−1 months)**	**AP1 (1 month)**	**AP2 (4 months)**	**AP3 (12 months)**	**AP4 (18 months)**
**Eligibilty screening**						
MMSE	●				●	
**Quality of life/subjective health**						
WHOQOL-BREF	●	●	●	●		●
WHOQOL-OLD	●	●	●	●		●
EQ5D-VAS	●	●	●	●		●
Qualitative Interview "Health and QOL"				●		
Focus groups "Health and QOL"						●
**Technology acceptance/usability**						
TAEG	●					
Usability			●		●	●
Qualitative interview "Technology Acceptance"				●		
Perceived system satisfaction				●		●
Technology estimates	●			●		
Focus groups "Technology Acceptance"						●
Frequency of Usage (technical analysis)						●
Visitation Protocols			●	●	●	●
Test calls and hotline contacts			●	●	●	●
**Other measurements**						
Sociodemographic Parameters	●					
Health threatening event in the past	●			●		●
Support for limitations	●			●		●

For evaluation of the outcomes a mixed-method approach with qualitative and quantitative methods is applied. Because of the heterogeneous combinations of technologies and participants, this modus operandi will provide the ability to gather additional information for qualitative analysis to the data collected for quantitative analysis.

Following measurements are performed:

Measurement for eligibility screening:

• MMSE: Cognitive function assessed by the German version [12] of the Mini Mental State Examination [13]

Measurements of health status and QOL:

• WHOQOL-BREF [14]: German version of the short form of the World Health Organization Quality of Life Questionnaire (Question nr. 21 was omitted due to the concerns of the consortium members)

• WHOQOL-OLD [15]: German version of the World Health Organization Quality of in the Elderly Questionnaire (Domains: “sensory”, “functions”, “autonomy”, “activities in the past, present and future”)

• EQ-5D VAS [16]: German Version of the Euroqol Visual Analogue Scale from the EQ-5D Quality of Life Questionnaire (The EQ-5D Questions 1–5 were omitted due to the concerns of the consortium members)

• Health and Quality of Life - Interviews: Semi-structured qualitative interviews and focus groups according to the bio-psycho-social model of Engel [17] and the multidimensional QOL-approach of the WHO [18]

Measurements of technology acceptance and usability:

• TAEG [19]: “Measuring technology affinity” questionnaire of the Technical University of Berlin.

• USABILITY Questionnaire developed by the usability competence team of the FH Technikum Wien based on: The System Usability Scale (SUS) [20], User Experience Questionnaire (UEQ) [21] and Microsoft Product Reaction Cards® [22]

• Technology acceptance - Interviews: Semi-structured qualitative interviews and focus groups according to the models of McCreadie&Tinker [23] for assistive technology for older people and the technology acceptance model(TAM) of Davis [24]:

• Perceived satisfaction question: “How many points you would give us for the ModuLAAr System?” (5-point Likert scale; AP2, AP4)

• Technology estimates:

What are your general estimates regarding the different modules (of the previous presentation)?” (open format, only at BA1)

“Which are the pros and cons you are estimating regarding the provided technologies?” (open format, only at BA1)

“Which estimates regarding the system had been met/had not been met?” (AP2, AP4)

• Frequency of system usage: The frequency how often the products had been used will be analyzed and reported anonymously (AP4)

• Visitation protocols: The visits will be documented in protocols that provide further input to relevant issues of the participants daily life and technology usage (AP3, AP4)

• Test calls and documentation of hotline contacts: Periodical phone calls are conducted by project staff to gather relevant information for issues arising with the provided technologies (AP1- AP4). Furthermore the participants’ hotline contacts are documented to identify for what reason the hotline was called (AP1- AP4).

Other Measurements:

• Socio-demographic parameters: sex, level of education, working experience, income, former housing conditions, chronic diseases, frequency of falls, previous health threatening events (BA1, AP2, AP4), level of social support, medicine intake.

• Support for limitations questions:

What are the most important physical limitations during the activities of daily living?” (open format)

“How important it is for you to get more support by doing your activities of daily living?” (5-point Likert scale)

“How important it is for you to get more support for your memory?” (5-point Likert scale)

“How secure do you feel in your environment/ flat?” (5-point Likert scale)

### Statistical analysis

Analyses will be conducted using SPSS® version 21.0 (SPSS Corp, Chicago, Illinois, USA) and Microsoft Excel 2010® software. Descriptive statistics and interferential statistics are performed. The sample data is carried out by frequencies or percentages, means, standard deviation, and graphics. Descriptive statistics are used to summarize demographic parameters and baseline data. General Linear Models (ANCOVA) controlling for baseline performance and other confounding variables is used if required. The delta-outcome of the baseline assessments (delta of BA1 to BA2) will be compared to the iterative delta of the data gathered by the intervention assessment (e.g. delta of AP1 to AP2, delta of AP1 to AP3) of each participant. To partially overcome the limitation of the lack of having a control group in the interim project analysis, the outcome data of the evaluated study population (delta of AP1 to AP2) is compared to those participants who finished the baseline assessment at that point in time (the same month) but are not yet provided with an installed system. All tests are two-sided at a significance level of p <0.05. T-test is used to compare baseline data and data of the follow-ups. Pearson and Spearman correlation coefficients are used for confounder identification. For the case that normal distributions couldn’t be reached non parametric tests (Wilcoxon, Mann-Whiteney-U-Test) are used in combination with Spearman and Kendall´s tau correlation coefficients.

### Qualitative analysis

The qualitative interviews and focus groups are digitally recorded and transcribed almost verbatim according to a rule set developed by Kuckartz [25]. If the participant doesn’t want the interview to be recorded, the main information of the interview will be documented in a protocol. Data analysis is performed based on the qualitative content analysis of Philipp Mayring [26].

### Triangulation of the results

The results of the analyzed quantitative data are compared to the results from the qualitative interviews and focus groups. This approach of method- triangulation [27] shall enable a more holistic view on the subject of research and provides the opportunity to partially overcome deficiencies of the investigators and/or methods.

## Discussion

This proposed study is designed to deliver new information to existing knowledge about AAL solutions regarding (a) QoL and health impacts on the end-users, and (b) aspects how technology acceptance and usability can be addressed and improved for complex ICT products for elderly.

The applied methodologies are in line with findings of official workshops [28] regarding AAL evaluations where, amongst others, the WHOQOL-Instruments as well as the EQ-5D were recommended to assess projects in this field of endeavor. By applying a mixed-method design to gather feedback or the participants and objective clinical results, we give the opportunity to overcome limitations of common standardized measurement tools which are designed according to a high-level overall approach to measure quality of life in different dimensions.

The potential of AAL is limited by a lack of evidence regarding possible impacts. It is strongly needed to assess how and which dimensions of QOL and health status of elderly people can be influenced by AAL solutions. In our study, usability of each user and aspects of technology acceptance are investigated because these parameters are closely related to the success of these socio-technical AAL systems and its impact on each individual.

Due to the framework-given, lack of having a control group in this study, the generalizability of our findings is limited to the treatment population, but the results will show if and how the impact of the applied AAL solutions on the elderly individuals is generated. The quasi-experimental design of our study allows us to make inferences on the effect of the intervention by looking at the changes of the baseline to post-test data/information. By interpreting these results, we can´t be sure that the differences in baseline-test and post-test are causally related to the effects of the ModuLAAr project. To cope partially with this fact, an in-depth view at the results, by applying triangulation of results of qualitative and quantitative methods is conducted. There also could be a volunteer bias generated during recruitment which affects the representativeness of the population but not the inferences of analyses. For future field trials, it is recommended to include a control arm to conclude the results to the general population which is investigated to enhance internal validity of those studies. Beside these limitations, the longitudinal study design in combination with a mixed-method approach is the best way to gather the needed information within the limited timeframe of the project and to describe effects and relationships of AAL in elderly in a sufficient way.

This longitudinal case-only field trial, involving a population aimed to recruit up to 50 participants, is the first explorative AAL- large scale study in Austria. The results will provide valuable information about QOL and health outcomes of AAL solutions and seeks to describe the pathways influencing the investigated parameters by advanced ICT considering aspects of technology acceptance and usability concerns of the individuals. It will provide a solid understanding to future research activities and evaluation studies in the field of AAL which aim to provide a holistic and user-centered AAL solution.

## Abbreviations

AP1: Assessment point1; AP2: Assessment point2; AP3: Assessment point3; AP4: Assessment point4; AAL: Ambient Assisted Living/Active and Assisted living; AALJP: Ambient Assisted Living Joint Program BA1, Baseline assessment point 1; BA2: Baseline assessment point 2; BMVIT: Austrian Federal Ministry for Transportation, Innovation and Technology; FFG: Austrian Research Promotion Agency; ICT: Information and communication technology; MMSE: Mini mental state examination; NFC: Near Field Communication; TAEG: Fragebogen zur Technikaffinität/Questionnaire for affinity to technologies; TAM: Technology acceptance model; QoL: Quality of Live; WHOQOL: World Health Organization Quality of Life.

## Competing interests

The authors declare that they have no competing interests.

## Authors’ contributions

CS is the main author of this manuscript. BP is the principle investigator of the study, designed the study together with CS. TD and MK advised on the study design and recommended appropriate measurements. BP, CS and JK are responsible for the elaboration and realization of the study. TD, MK and JK advised on editing and corrected the manuscript. All authors have read and approved the final version of the manuscript.

## Authors’ information

CS: biomedical scientist, research associate at the Biomedical Systems Division, Department of Health and Environment, AIT Austrian Institute of Technology and PhD-student at the Institute of Social Medicine, Centre for Public Health, Medical University of Vienna.

BP: scientist at the Biomedical Systems Division, Department of Health and Environment, AIT Austrian Institute of Technology.

JK: scientist and project manager at the Biomedical Systems Division, Department of Health and Environment, AIT Austrian Institute of Technology.

MK: medical doctor, professor and head of the Institute of Environmental Medicine, Centre for Public Health, Medical University of Vienna.

TD: medical doctor, associate professor at the Institute of Social Medicine, Centre for Public Health, Medical University of Vienna.

## Pre-publication history

The pre-publication history for this paper can be accessed here:

http://www.biomedcentral.com/1471-2458/14/736/prepub
